# A palladium bio-nanocomposite as an efficient heterogeneous catalyst for nitro reduction: a fungus mediated green and sustainable process

**DOI:** 10.1039/d6na00174b

**Published:** 2026-05-28

**Authors:** Hemal B. Rathod, Amar G. Deshmukh, Yashasvi N. Desai, Paresh N. Patel

**Affiliations:** a Laboratory of Bio-Organic Chemistry, Tarsadia Institute of Chemical Science (TICS), Uka Tarsadia University Bardoli – 394 350 Gujarat India pareshn111@yahoo.com

## Abstract

Metallic nanocatalysts such as palladium nanoparticles (Pd-NPs) possess remarkable catalytic activity owing to their high surface-to-volume ratio; however, aggregation and metal leaching significantly compromise their stability and practical applicability. In this work, we report a facile and sustainable biosynthetic route for the preparation of bio-stabilized Pd-NPs using the fungal strain *Aspergillus trinidadensis* VM ST01 (OL587588) as a green reducing and capping agent. The strategy enables simultaneous bio-reduction of Pd^2+^ ions, nucleation and *in situ* surface functionalization without the use of hazardous chemicals, surfactants, buffer, or external stabilizers. The influence of culture age (24–54 h), biomass loading (0.08–0.24 g mL^−1^), and incubation time (8–24 h) on NP formation was systematically investigated to achieve controlled synthesis. Comprehensive physicochemical characterization (UV-vis, FT-IR, XRD, SEM, TEM, elemental mapping, EDX, XPS, and TGA) confirmed the formation of uniformly distributed Pd-NPs with an average size of ∼35 nm, embedded and stabilized within the fungal biomass matrix. The bio-organic framework surrounding the nanoparticles effectively suppresses aggregation and minimizes palladium leaching, thereby enhancing catalyst durability. The catalytic performance of the biosynthesized AtPdNPs was evaluated for the hydrogenation of nitro-benzene (NB) to amino-benzene (AB) as a model reaction under mild and aqueous conditions (NaBH_4_, ambient temperature and ambient pressure). The optimized catalyst (0.16 g mL^−1^ biomass, 36 h of culture age, and 24 h of incubation) achieved complete conversion within 30 min, delivering a turnover frequency (TOF) of 832 h^−1^ and a turnover number (TON) of 416. The kinetics for the reduction of NB to AB was investigated, and the catalytic activity of AtPdNPs was evaluated as the pseudo-first-order rate constant (*k*_app_). The *in situ* bio-coating of AtPdNPs significantly reduces palladium leaching (<1.5% ppm), enhances storage stability and makes it an environmentally compatible material. As a result, the catalyst maintains its structural integrity and catalytic performance under industry relevant conditions.

## Abstract 2

Palladium nanoparticles exhibit excellent catalytic activity due to their high surface area; however, aggregation and metal leaching reduce stability and recyclability, which can be overcome by forming bio-nanocomposites.

## Graphical abstract

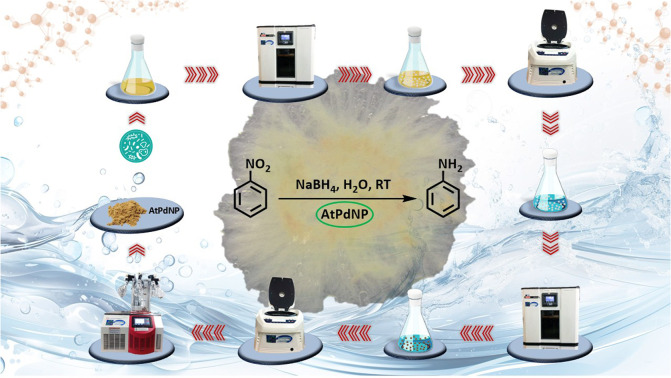

## 1. Introduction

**Fig. 1:**
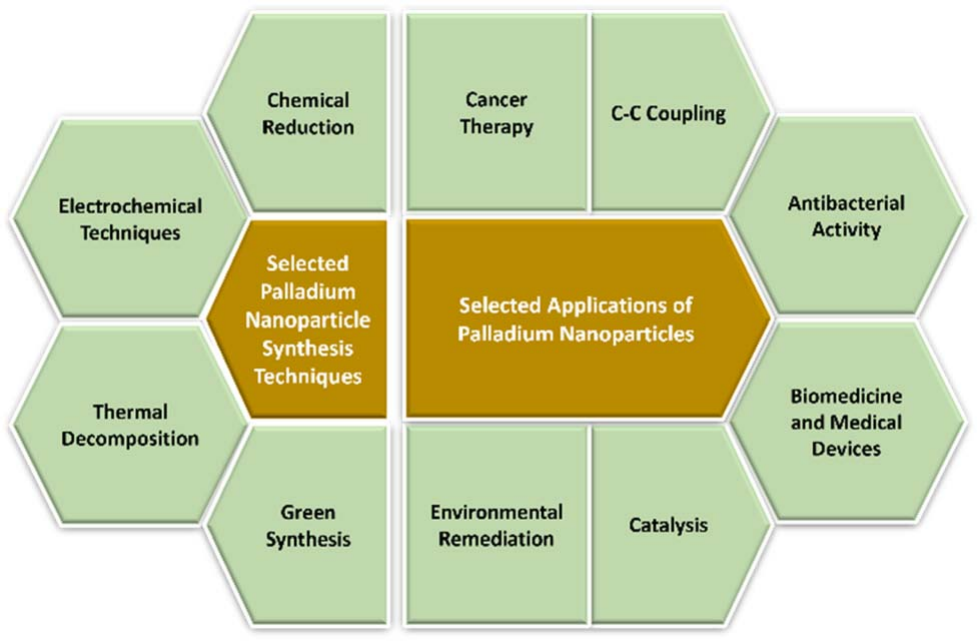
Selected techniques for synthesis and applications of palladium nanoparticles.

Materials incorporating palladium play a dynamic role in diverse research areas, including water purification,^1,2^ electrochemistry,^3,4^ drug delivery,^5^ sensing,^6^ and organic transformations.^7–9^ In particular, palladium nanoparticles (Pd-NPs) have attracted significant attention owing to their enhanced physicochemical and optical properties at the nanoscale.^10–12^ The high surface-to-volume ratio and abundant active sites of Pd-NPs make them highly efficient catalysts, especially in reduction reactions and carbon–carbon coupling processes.^13–16^ Beyond synthetic chemistry, Pd-NPs are widely applied in environmental remediation for pollutant degradation and exhaust gas treatment.^17^ Their antimicrobial activity, arising from reactive oxygen species (ROS) generation and membrane disruption, has also enabled applications in medical devices, drug delivery systems, diagnostics, and photothermal cancer therapy (Fig. 1).^18,19^

Conventionally, Pd-NPs are synthesized *via* chemical reduction,^20^ electrochemical deposition,^21^ and thermal decomposition methods.^22,23^ Although these approaches can produce high-quality nanoparticles, they frequently require toxic reducing agents, stabilizers, or organic ligands. Such processes may generate hazardous by-products, limit large-scale sustainability, and leave residual surface contaminants that adversely affect the catalytic and biological performance.^24^ Moreover, nanoparticle aggregation remains a persistent challenge, leading to diminished catalytic efficiency and stability.^25^ Currently, the growing interest in sustainable and environmentally friendly processes has led to the development of green synthetic methods for PdNPs^26^ (Fig. 1).

In response to increasing environmental concerns, green and sustainable synthetic methodologies for Pd-NPs have gained considerable attention.^27,28^ Several publications in the literature describe the production of metal nanoparticles (NPs) using different biomaterials which emphasize the safe, non-toxic and ecologically friendly character.^29–33^ The integration of nanotechnology and biotechnology, often termed nanobiotechnology, offers innovative pathways for environmentally benign nanomaterial fabrication.^34–36^ Biogenic synthesis using plant extracts, bacteria, or fungi provides a promising alternative to conventional physicochemical routes.^37,38^ These biological systems contain naturally occurring biomolecules capable of acting simultaneously as reducing and stabilizing agents, enabling nanoparticle formation under mild conditions without the need for external toxic chemicals.^39–41^ Importantly, *in situ* bio-functionalization can minimize aggregation and eliminate the necessity for additional capping agents. Strictly, “green” methods can be used to synthesize nanoparticles by using environmentally friendly solvent systems and safe reducing and stabilizing agents.^42,43^ Green synthesis of Pd-NPs uses natural reducing agents like plant extracts, bacteria, or fungi to reduce palladium salts, making it environmentally friendly, biocompatible, and have unique properties. These methods are cost-effective, sustainable, and reduce environmental impact.^44^ Plant extracts contain bioactive compounds that act as reducing and capping agents, resulting in stable, uniformly dispersed nanoparticles. Microbial synthesis using bacteria and fungi offers an eco-friendly alternative, producing distinct morphological and functional nanoparticles under mild conditions.^45^ Nevertheless, impact of an additional capping agent or stabilizer can easily be avoided due to *in situ* stabilization of the catalyst by the bio-molecules tethered to the metal centre.^46,47^ Fungal systems are particularly attractive due to their high metal tolerance, rich enzymatic machinery, and abundant extracellular proteins that facilitate controlled nanoparticles formation and stabilization.^27,38^

Inspired by these advantages and in continuation of our efforts toward sustainable nanoparticles development,^27,37^ we herein report a green biosynthetic strategy for the preparation of bio-stabilized Pd-NPs. The synthesized catalyst demonstrates excellent performance in the reduction of nitrobenzene to aniline under environmentally benign conditions, delivering high turnover frequency (TOF) and turnover number (TON). This study highlights a sustainable and biologically integrated approach for designing efficient Pd-based heterogeneous nanocatalysts.

## 2. Experimental

### 2.1 Chemicals and characterization techniques

Palladium(ii) chloride (Reagent Plus®, 99%) was purchased from Sigma-Aldrich. Nitrobenzene, aniline, acetonitrile (HPLC), and H_2_O (HPLC) were purchased from Rankem. Potato dextrose broth (granulated) and potato dextrose agar were purchased from Hi-Media. Laboratory-prepared double-distilled water was used throughout the experiment.

UV-vis spectroscopy (Shimadzu UV-1900i), zeta size and zeta potential measurements (Malvern Zetasizer Lab), Fourier Transform Infrared (FT-IR) spectroscopy (Bruker ALPHA II), Powder X-ray Diffraction (PXRD) (Malvern Panalytical X'Pert Pro), Energy Dispersive X-ray (EDX) spectroscopy, X-ray Photoelectron Spectroscopy (XPS) (Shimadzu Axis Supra Kratos Ultra-II), Scanning Electron Microscopy (SEM), Transmission Electron Microscopy (TEM), Thermogravimetric Analysis (TGA) and Differential Scanning Calorimetry (DSC) (Mettler Toledo), and High Performance Liquid Chromatography (HPLC) (Shimadzu Prominence-i LC-2030 Plus) were performed.

### 2.2 Isolation and growth of *Aspergillus trinidadensis* VM ST01′ OL587588 (At) (fungi)

*Aspergillus trinidadensis* VM ST01′ OL587588 (At) (fungus) was selected for this study. It was extracted from the soil of our campus garden and cultivated with the support of our Microbiology Department. Optic microscopy and lactophenol cotton blue staining were used to examine the strain's morphology. This fungal strain's pure culture was maintained on potato dextrose agar slants.^27^

Potato dextrose agar (39.0 g) was dissolved in one liter of distilled water. The resultant solution was heated at its boiling point to completely dissolve the media. Later on, it was sterilized using an autoclave set to the validated cycle, or for 15 minutes at 121 °C and 15 kg cm^−2^ pressure. Then, the temperature was reduced between 40 and 45 °C. After blending well, it was transferred to sterilized Petri dishes and incubated at 36 °C for 72 h. Every month, the slants were prepared and maintained.

After this, the primary media was prepared by dissolving 24 g of potato dextrose broth in one litre of distilled water. Then, it was sterilized in an autoclave at 15 kg cm^−2^ pressure and 121 °C for 15 minutes. After that, the culture from the agar slant was charged at room temperature, about 25 °C, and incubated for different time intervals at 32 °C and 150 rpm. The resultant bio-mass was separated by using a centrifuge set at 3000 rpm for 5 minutes, and media was removed from the biomass. It was washed with sterile double-distilled water, the entire process of centrifugation was repeated three times and biomass (fungi) was collected.

### 2.3 Palladium nanoparticle (AtPdNP) synthesis using *Aspergillus trinidadensis* (At)

**Fig. 2:**
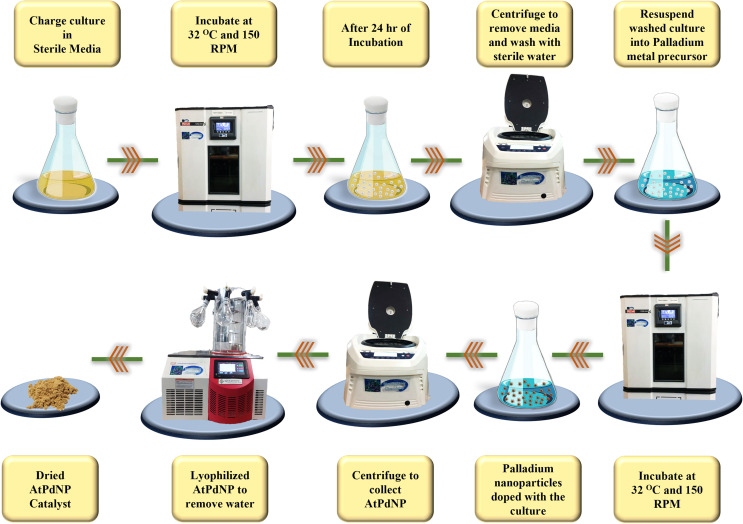
Graphical representation of procedure for AtPdNP synthesis.

A 1 M stock solution of palladium(ii) chloride (PdCl_2_) in 100 mL sterile distilled water (metal precursor) was prepared. Then, a wet biomass (4 g of 48 h aged culture) was re-suspended in 25 mL metal solution with a concentration of 1 mM. The obtained solution was incubated for 24 h at 32 °C and 150 rpm. The resultant bio-nano-composite (AtPdNP) was centrifuged for five minutes at 3000 rpm and 25 °C. The process of centrifugation was repeated for washing with distilled water. Finally, the palladium nanoparticles doped with biomass (AtPdNP) were dried using a lyophilizer (Fig. 2). A reference sample devoid of biomass and an additional sample devoid of metal solution were also prepared under the same conditions as a controlled experiment.

As an optimisation process, several culture ages (24, 30, 40, 48, and 55 h) were used with a fixed biomass concentration (160 mg mL^−1^), metal concentration (1 mM) and 24 h of incubation for AtPdNP synthesis. UV-vis spectroscopy and catalytic activity study of the prepared AtPdNP were performed to determine the optimal culture age for the process. As a second optimization, various biomass concentrations (80 mg mL^−1^, 120 mg mL^−1^, 160 mg mL^−1^, 200 mg mL^−1^, and 240 mg mL^−1^) were used with 24 h culture age, 1 mM metal concentration and 24 h of incubation. Here also, UV-vis spectroscopy and catalytic activity study of the prepared AtPdNP were used to determine the optimal biomass content. The production of palladium nanoparticles was further optimised by varying the incubation time (8, 16, or 24 h) but with fixed other parameters, *i.e.*, 24 h age culture, 120 mg mL^−1^ bio-mass and 1 mM metal solution concentration. UV-vis spectroscopy and catalytic activity study of AtPdNPs were then used to determine the optimal incubation time for synthesis.

### 2.4 Process development for nitro reduction with AtPdNP

**Scheme 1:**
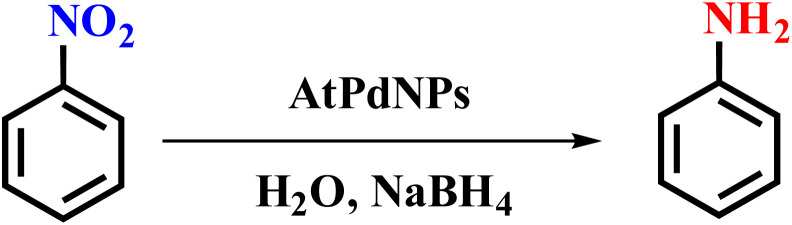
Reduction of nitrobenzene using AtPdNP.

Application of the prepared AtPdNP was demonstrated for catalytic hydrogenation. As a model example, the process for hydrogenation of nitrobenzene was developed. The dried catalyst AtPdNP (5 mg) was re-suspended in 5 mL of double-distilled water, and 1 mM (0.102 mL) nitrobenzene was added into 25 mL of RBF. The RBF was then placed in an ice bath and kept at a temperature between 0 and 5 °C. Afterward, 0.075 g (2 mM) of NaBH_4_ was added as a hydride source, and the reaction flask was tightly sealed to prevent gas leaking. After 5 min, the ice bath was removed and the resultant solution was stirred at room temperature. Thin-layer chromatography and ninhydrin staining were used to monitor the reaction. Once the reduction was finished, 5 mL of ethyl acetate was added and mixed thoroughly. Then, it was allowed to settle under stable conditions to extract the product and separate the layers. After separating the organic phase and drying it with sodium sulphate anhydrous, ethyl acetate was evaporated to collect the final product (Scheme 1). The residual catalyst was washed properly with water and reused for the second batch.

### 2.5 Kinetic study for nitro reduction with AtPdNP

**Fig. 3:**
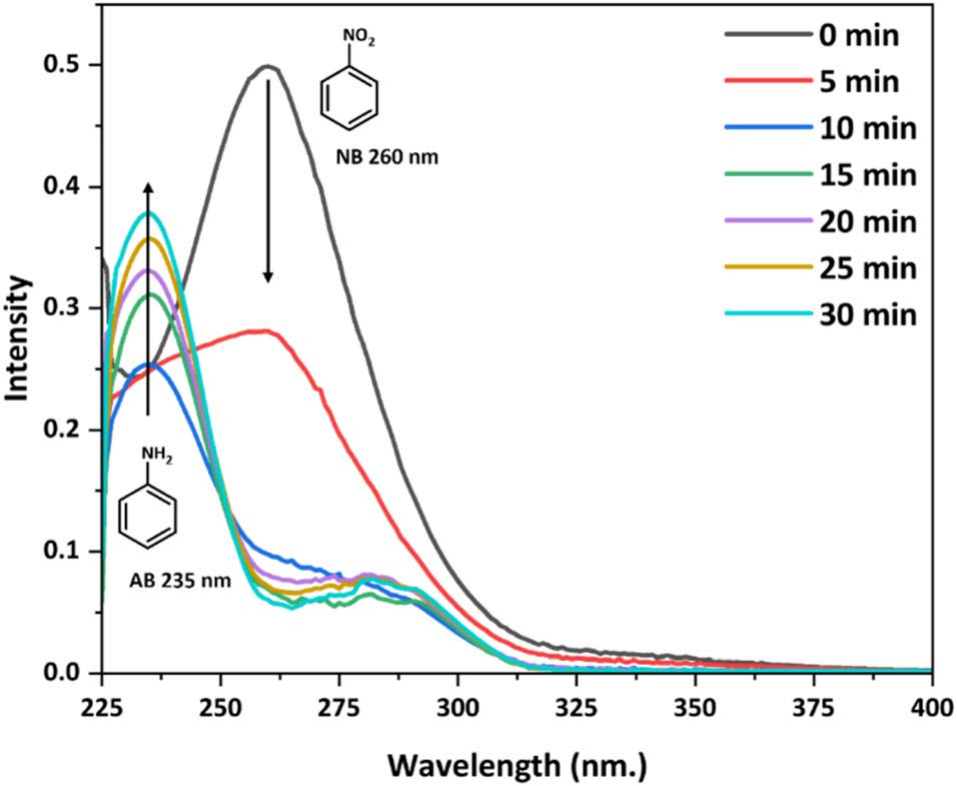
NB to AB reaction study by UV-vis.

To perform the kinetic study, conversion of nitro-benzene (NB) to amino-benzene (AB) was quantitatively analysed using spectrophotometry. Initially, the maximum absorption (*λ*_max_) was recorded at 260 nm for NB, and after completion of the reaction it was gradually shifted to 235 nm for AB. The absorption peak at 260 nm decreased with time, indicating the reduction of NB (Fig. 3).

**Fig. 4:**
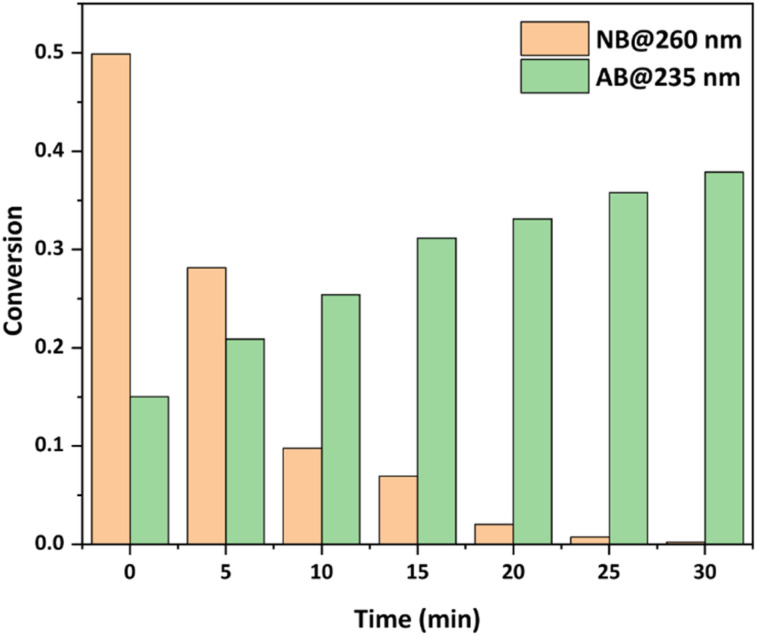
Conversion of NB to AB based on UV-vis absorbance.

The pseudo-first-order rate constants (*k*_app_) were calculated by fitting the first-order rate constant equation for ln(*A*_0_/*A*_*t*_) values against *t*(s) with a linear fit, where *A*_0_ and *A*_*t*_ are the initial absorbance and absorbance after a given time, respectively. The correlation coefficients, *i.e.*, *r* values for this linear fit, varied from 0.96 to 0.99. The *k*_app_ values were obtained with an accuracy of ±5%. The experimental *k*_app_ values are the average values for triplicate experiments at a temperature of 298.15 K (Fig. 4).

### 2.6 Studies on the pH, storage stability and catalyst leaching

To study the pH stability of the AtPdNP catalyst, aqueous solutions with different pHs were prepared. Double distilled water was utilised as the primary medium; HCL was employed for its acidic nature, while NaOH was utilised for its basic nature. After making solutions of pH 2, 4, 6, 8, 10 and 12, the catalyst AtPdNP was re-suspended in different pH solutions and allowed to sit for a day. After 24 h, its efficiency was evaluated by study of its catalytic activity and UV-vis spectroscopy to make sure there had not been any noticeable change. To determine the storage stability of the AtPdNP catalyst, the catalyst sample was stored for six months under ambient conditions. The AtPdNP exhibited storage stability as well by showing no discernible changes in catalytic efficiency after six months. The leaching of AtPdNP catalyst was studied under three different conditions by suspending 50 mg catalyst in 5 mL distilled water. The resultant A-solution was stirred for 24 h, B-solution was sonicated at 80 °C for 6 h, and C-solution was used for nitro reduction with sodium borohydride for 1 h. Later, the catalyst was separated by filtration, and the filtrate solutions were used for ICP-OES analysis (Table 1). Detailed calculations are given in the SI. The obtained ICP-OES results clearly demonstrated the negligible leaching of palladium in all three filtrates (*i.e.* less than 1.15% ppm). This clearly confirmed the heterogeneous nature of the prepared catalyst.

**Table 1:** Catalyst leaching study by ICP-OES

Sample code	Pd in AtPdNP (ppm)	Pd leaching (ppm)	Pd leaching (% ppm)
A	530	3.15	0.59
B	530	5.99	1.13
C	530	6.03	1.13

### 2.7 Characterization methods

UV-vis spectroscopy was used as a primary tool to evaluate the catalyst using the absorbance differences between standard palladium(ii) chloride (PdCl_2_), standard biomass (At) and the synthesized AtPdNP, covering a 200–800 nm spectral range. All samples were prepared in double-distilled water (DDW). The AtPdNP samples were re-suspended in DDW, homogenized with a homogenizer and sonicated for one hour to ensure proper dispersion. The zeta size and zeta potential for these samples were analysed. Further characterization of lyophilized powder was carried out using FT-IR spectroscopy, powder XRD, EDX, and XPS. The XPS measurements employed monochromatic Al Kα radiation (1486.6 eV) with an operating power of 50 W (15 kV). The analysed area was set to 300 µm. Survey spectra were recorded with a pass energy of 117.4 eV and an energy step of 0.125 eV, covering a spectral range of 400–2500 eV. For detailed atomic element analysis, a pass energy of 23.5 eV and an energy step of 0.025 eV were applied. The take-off angle was maintained at 25° relative to the sample substrate. To prevent charging during XPS analysis, the samples were neutralized using both low-energy ion and electron beams. SEM, TEM, TGA, and DSC were performed to further characterize the dried AtPdNP, providing insights into their morphology, thermal stability and composition. HPLC was conducted for monitoring the reduction reaction and product characterisation.

## 3. Results and discussion

### 3.1 Culture age effects on synthesis of AtPdNP and its catalytic activity

**Fig. 5:**
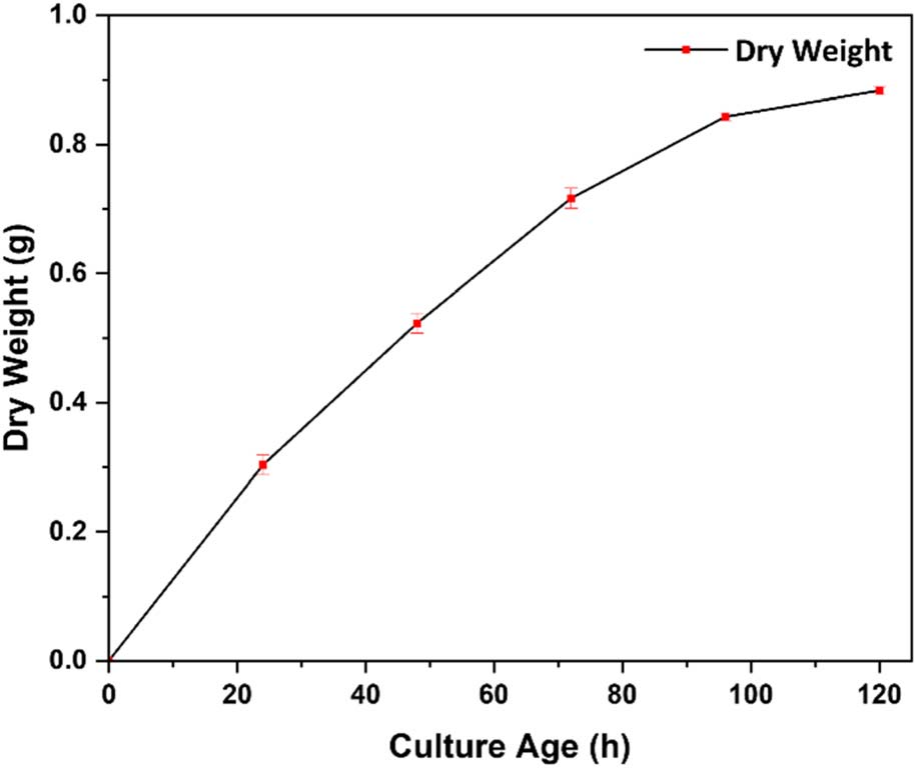
Growth curve of *Aspergillus trinidadensis* VM ST01′ OL587588.

*Aspergillus trinidadensis* VM ST01′ OL587588 (fungus) exhibits the following growth behaviors on potato dextrose broth media: a 0–24 h lag phase, a 24–96 h log phase, a 96–120 h stationary phase, and a decline phase after 120 h (Fig. 5).

The best time to use this biomass for the synthesis of palladium nanoparticles was between 24 and 96 h, when the biomass was in the log phase and more enzymes were released by the culture, facilitating the synthesis of nanoparticles. Within this age range, the synthesis of palladium nanoparticles was investigated at 24, 30, 36, 42, 48, and 54 h. According to the catalytic activity study, AtPdNP prepared with 36 h culture age converted nitrobenzene to aniline 100% within half hour, making 36 h the ideal culture age (Table 2).

**Table 2:** Nitrobenzene reduction using the prepared AtPdNP with varying (At) culture ages

Culture age (h)	Biomass weight (g mL^−1^)	Conversion (%)
24	0.160	94
30	0.160	98
36	0.160	100
42	0.160	98
48	0.160	95
54	0.160	87

The 36 h culture age is the best option for catalytic applications because it exhibits the highest efficiency among all the studied culture ages. All these experiments were performed in triplicate, and average results are presented here.

### 3.2 Effect of biomass quantity on catalytic activity and doping of nanoparticles

Different biomass amounts with a 36 h culture age were used to optimise the biomass quantity for AtPdNP synthesis. Biomass amounts ranging from 0.080 g mL^−1^ to 0.240 g mL^−1^ were used with a constant palladium concentration of 1 mM. The highest catalytic activity was with biomass concentrations of up to 0.160 g mL^−1^. Beyond this, there was no noticeable increase in catalytic performance, maybe due to low palladium uptake with increasing biomass quantity (Table 3). Thus, 0.160 g mL^−1^ biomass concentration was used for the remaining optimisation studies. All experiments were performed in triplicate, and average results are presented here.

**Table 3:** Nitrobenzene reduction using the prepared AtPdNP with varying biomass quantities

Culture age (h)	Biomass (g mL^−1^)	Conversion (%)
36	0.080	100
36	0.120	100
36	0.160	100
36	0.200	95
36	0.240	80

### 3.3 Optimising the incubation period for synthesis of AtPdNP and its catalytic activity

In this experiment, the optimal culture age of 36 h and biomass amount of 0.160 g mL^−1^ were used, and the incubation time for the synthesis of palladium nanoparticles were varied to 8, 16 and 24 h. As per the obtained results, the best catalytic activity was observed with the catalyst prepared with 24 h of incubation time (Table 4). All these studies were performed in triplicate. and average results are presented here.

**Table 4:** Nitrobenzene reduction using the prepared AtPdNP with varying incubation times

Culture age (h)	Biomass (g mL^−1^)	Incubation time (h)	Conversion (%)
36	0.160	8	30
36	0.160	16	50
36	0.160	24	100

### 3.4 Controlled experiments for nitrobenzene reduction

A series of control experiments were conducted to gain a comprehensive understanding of the reaction mechanism and the role of each component. These studies confirmed the critical contribution of each component to the overall reduction process (Table 5).

**Table 5:** Nitrobenzene (NB) reduction using the prepared AtPdNP (controlled reaction)

NB (mmol)	Pd (µmol) AtPdNP	NaBH_4_ (mmol)	Reaction time	Conversion (%)
1	—	—	4 h	—
1	Blank At	—	4 h	—
1	Blank At	2	4 h	—
1	5	—	4 h	—
1	—	2	4 h	—
—	5	2	4 h	—
**1**	**5**	**2**	**4 h**	**100**

### 3.5 Kinetic analysis of nitrobenzene (NB)

Due to the presence of excess NaBH_4_ compared with NB, the calculated rate constant of the reaction was a pseudo first-order rate constant (*k*_app_). The apparent rate constant was calculated using eqn (1) for a first-order reaction.^38^

1 ln[*A*_*t*_/*A*_0_] = −*k*_app_*t*

where *A*_0_ and *A*_*t*_ are the absorbance of nitro compounds at the initial time and time *t* in second (s), respectively, and *k*_app_ is the apparent rate constant.

**Fig. 6:**
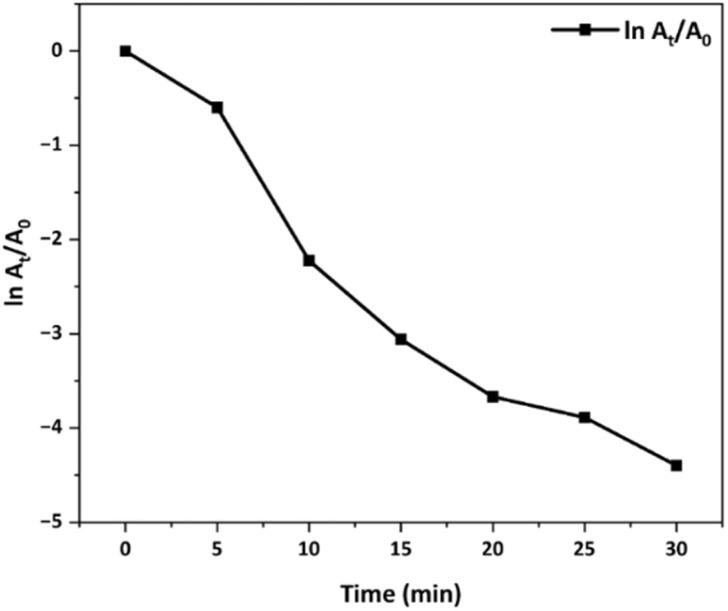
Pseudo-first-order kinetics of NB to AB conversion.

The reaction kinetics was examined by measuring the *k*_app_ values for the reduction of NB. The rate of reaction was determined by measuring the decrease in the absorption intensity at *λ*_max_ = 360 nm of NB with time for the reduction. The *k*_app_ was calculated from the slope obtained by plotting ln(*A*_*t*_/*A*_0_) *vs.* time (Fig. 6). All these measurements were performed in triplicate, and average results are presented here.

Finally, a comparison of heterogeneous palladium nanoparticle catalysts, prepared using different techniques and those reported in the literature (Table S4), indicates that the present catalyst is a highly efficient and promising alternative.

### 3.6 Characterization of the prepared AtPdNP

#### 3.6.1 Ultraviolet-visible spectroscopy (UV-vis)

**Fig. 7:**
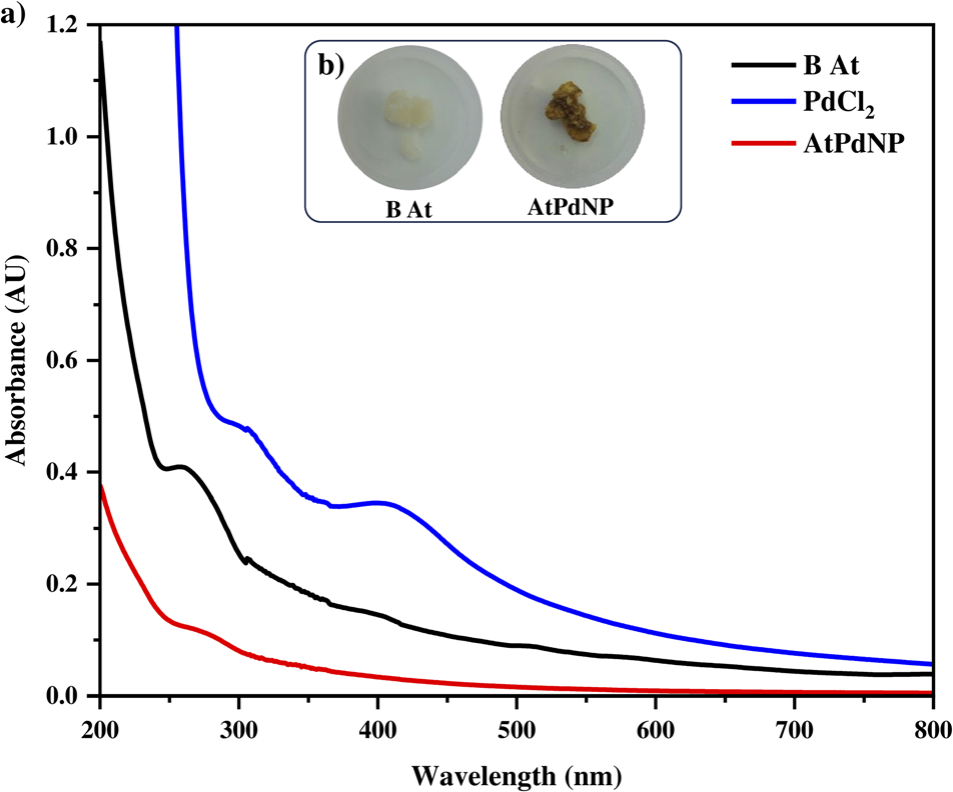
(a) UV-vis spectra of PdCl_2_, B At, and AtPdNP. (b) Visual analysis of B At and AtPdNP.

UV-vis techniques were used to analyse the synthesis of palladium nanoparticles. Following the synthesis of palladium nanoparticles, the colour of the biomass changed from pale white to brown (Fig. 7b). In order to prepare the sample, it had to be re-suspended in double-distilled water (DDW), homogenised using a homogeniser, sonicated for an hour, and then taken for UV-vis examination.

Because of the protein, the blank biomass B-At has an absorbance of 264 nm; on the other hand, blank palladium(ii) chloride has an absorbance of 411 nm. The absorbance of palladium(ii) chloride vanished following the synthesis of nanoparticles, as observed in the AtPdNP sample (Fig. 7a).

#### 3.6.2 Fourier transform infrared spectroscopy

**Fig. 8:**
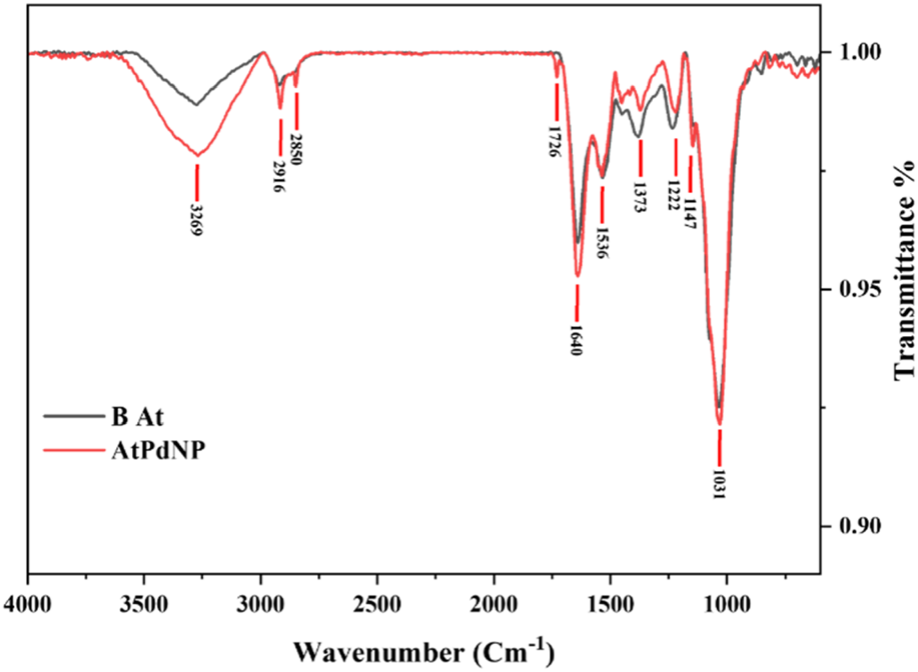
FT-IR spectra of B-At and AtPdNP.

To investigate the chemical composition and functional group involvement in both the native biomass (B At, *Aspergillus trinidadensis* VM ST01) and palladium nanoparticle-incorporated biomass (AtPdNP), FT-IR spectroscopy was employed as a key analytical tool. The spectra provide valuable insights into the interaction between biomolecular functional groups and metal nanoparticles (Fig. 8). In the case of the blank biomass (B At), the absorption band at 1031 cm^−1^ is attributed to C–N stretching vibrations, which are typically associated with aliphatic amines present in proteins and other nitrogen-containing biomolecules. The presence of multiple bands at 1222, 1373, 1536, and 1640 cm^−1^ further supports the contribution of amide and amine functionalities. Specifically, the band around 1536 cm^−1^ can be assigned to N–H bending (amide II), while the band near 1640 cm^−1^ corresponds to C=O stretching vibrations of amide I groups, indicating the presence of peptide linkages in fungal proteins. The peaks at 2916 cm^−1^ and 3269 cm^−1^ are attributed to aliphatic C–H stretching and N–H/O–H stretching vibrations, respectively, suggesting the presence of polysaccharides, proteins, and hydroxyl-rich biomolecules within the biomass matrix. Upon doping with palladium nanoparticles (AtPdNP), the FT-IR spectrum retains most of the characteristic bands of the native biomass, confirming that the fundamental biochemical framework remains intact. However, significant changes in peak sharpness, intensity, and slight shifts in wavenumber are observed. These variations indicate strong interactions between palladium nanoparticles and the functional groups of the biomass. Such shifts are commonly associated with coordination or binding of metal nanoparticles with electron-donating groups such as amines, hydroxyls, and carbonyls. Notably, the appearance of new bands at 1147, 1726, and 2850 cm^−1^ in the AtPdNP spectrum provides further evidence of structural modification. The band at 1147 cm^−1^ may be attributed to C–O stretching vibrations, possibly arising from polysaccharide or alcohol groups involved in nanoparticle stabilization. The prominent band at 1726 cm^−1^ corresponds to C=O stretching of carbonyl or ester groups, suggesting oxidation or modification of functional groups during nanoparticle formation. The band at 2850 cm^−1^ is assigned to symmetric C–H stretching of aliphatic chains, indicating alterations in lipid or hydrocarbon environments. These spectral changes strongly suggest that biomolecules present in the fungal biomass—such as proteins, polysaccharides, and other metabolites—play a dual role as a reducing as well as a stabilizing (capping) agent during the formation of palladium nanoparticles. The interaction between PdNPs and functional groups leads to changes in the vibrational environment, thereby confirming the successful incorporation of nanoparticles into the biomass matrix.

Overall, the FT-IR analysis clearly demonstrates that palladium nanoparticle doping induces significant chemical interactions and structural modifications in the biomass, without completely disrupting its inherent biochemical architecture. This supports the hypothesis that the biosynthesized PdNPs are effectively stabilized by the functional groups present in *Aspergillus trinidadensis* biomass, which is crucial for their potential catalytic and environmental applications.

#### 3.6.3 Zeta size and zeta potential measurements

The dynamic light scattering (DLS) analysis revealed that the synthesized palladium nanoparticles exhibited an average hydrodynamic diameter of 141.8 ± 1.9 nm with a polydispersity index (PDI) of 1.000, indicating moderate size uniformity in the colloidal suspension. The zeta potential was measured to be −0.472 mV, suggesting a negative surface charge and good colloidal stability due to electrostatic repulsion. These measurements were carried out at 25 °C in aqueous medium (Fig. S1, S2 and Tables S1, S2).

#### 3.6.4 Thermogravimetric analysis (TGA) & differential scanning calorimetry (DSC)

**Fig. 9:**
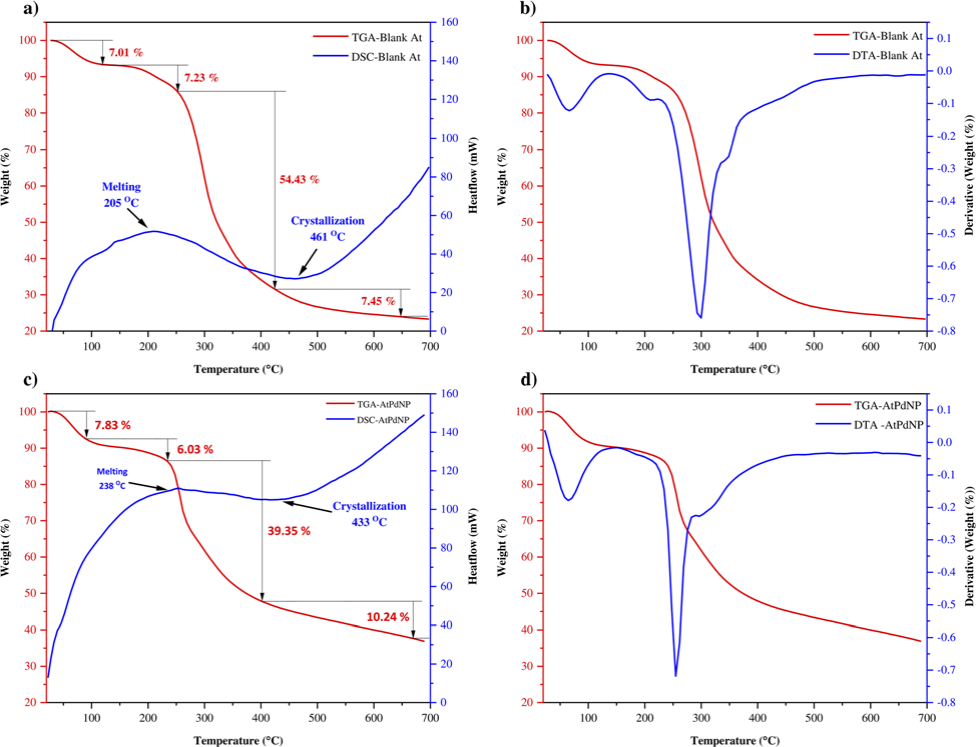
(a) TGA-DSC of blank At, (b) TGA-DTA of blank At, (c) TGA-DSC of AtPdNP, and (d) TGA-DTA of AtPdNP.

The thermal stability, composition, decomposition temperatures, moisture content, melting point, crystallisation point, and thermal events of AtPdNP were examined using thermogravimetric analysis (TGA), differential scanning calorimetry (DSC), and differential thermal analysis (DTA), as shown in Fig. 9. The initial weight loss for the moisture in blank At was 7.01% up to 119 °C (9a). After that, the initial sample decomposed from 119 °C to 252 °C, which was 7.23%.

The sample's major decomposition occurred at a faster rate up to 425 °C, 54.43%, and the final decomposition was slower up to 647 °C, which was 7.25%. The total decomposition of the sample was 75.92%, leaving carbon ash at the end. Biomass melting occurred at 205 °C, and it crystallized at 461 °C. The thermal analysis of blank At revealed two distinct thermal events (9b). The first event, associated with the initial decomposition of moisture, exhibited a broad and shallow peak at 65 °C. The second major thermal event occurred at 297 °C, where the sample underwent significant decomposition. The initial weight loss for the moisture in AtPdNP up to 91 °C was 7.83% (9c). After that, the initial sample decomposed from 91 °C to 234 °C, which was 6.03%. The sample's major decomposition occurred at a faster rate up to 400 °C, 39.35%, and the final decomposition was slower up to 670 °C, which was 10.24%. The total decomposition of the sample was 63.45%, leaving carbon ash and palladium oxide at the end. The AtPdNP melting occurred at 238 °C, and it crystallized at 433 °C. The thermal analysis of AtPdNP also revealed two distinct thermal events (9d). The first event, associated with the initial decomposition of moisture, exhibited a broad and shallow peak at 62 °C. The second major thermal event occurred at 255 °C, where the sample underwent significant decomposition.

#### 3.6.5 X-ray photoelectron spectroscopy (XPS)

**Fig. 10:**
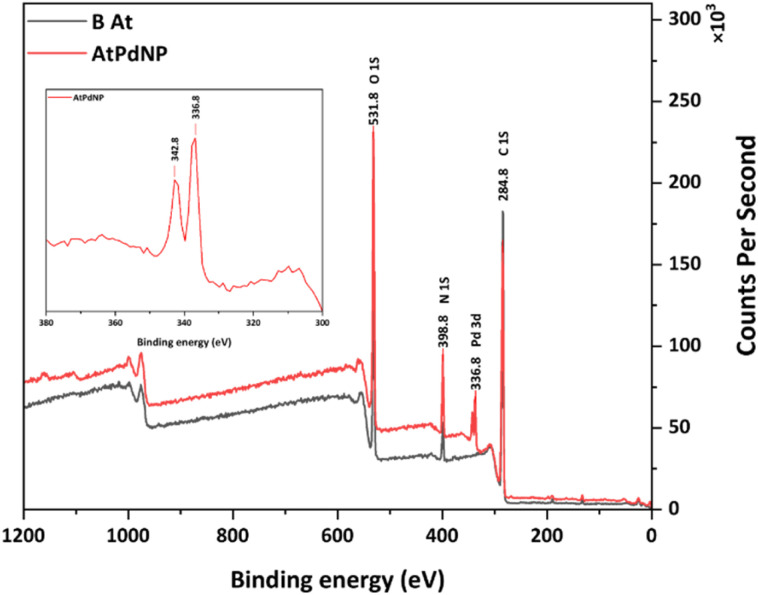
XPS spectroscopy of B-At and AtPdNP.

XPS measurements were performed to determine the composition present in the blank sample of *Aspergillus trinidadensis* VM ST01 biomass (B-At) and biomass doped with PdNP (AtPdNP). Binding energies for carbon 1s at 284.8 eV, nitrogen 1s at 399.8 eV, and oxygen 1s at 532.8 eV were observed in the B-At. However, in the case of AtPdNP, two more binding energies at 336.80 eV and 342.80 eV for Pd0 corresponding to Pd3d_5/2_ and Pd3d_3/2_, respectively, were observed (Fig. 10).^17^ These energies are relatively high for Pd0, which may be due to the interaction between Pd NPs and the oxygen/nitrogen containing groups within lignin during the carbonization process.^18^ Also, it is noted that the Pd 3d spectrum of Pd-0.3 sample showed no peaks corresponding to Pd^2+^ at 338.00 eV and 343.30 eV, respectively, and indicated the absence of Pd^2+^ species. These results confirmed the successful reduction and nucleation of Pd NPs in one-pot synthesis.

#### 3.6.6 Powder X-ray diffraction

**Fig. 11:**
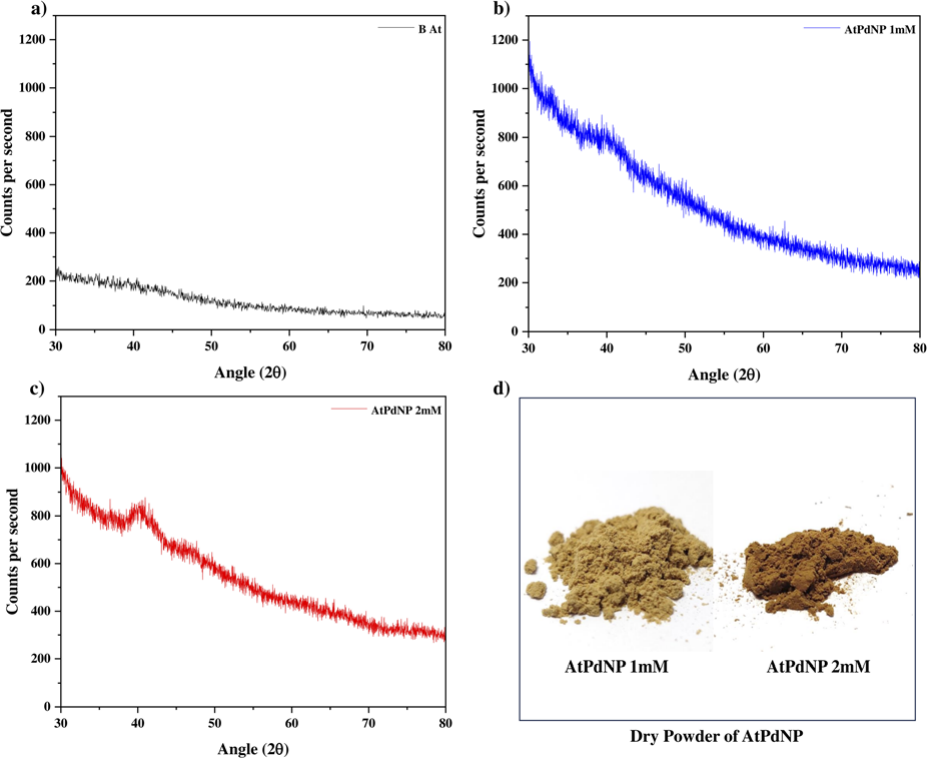
(a) Powder XRD of blank At, (b) powder XRD of 1 mM AtPdNP, (c) powder XRD of 2 mM AtPdNP, and (d) visual difference between 1 mM AtPdNP and 2 mM AtPdNP.

Powder XRD was used to analyse the crystalline nature of the AtPdNP catalyst. Since palladium was not doped into the culture, blank At biomass was used as a reference (Fig. 11a). AtPdNP prepared with 1 mM palladium chloride (Fig. 11b) was used for powder XRD analysis. A small but very sharp hump-shaped peak was observed between 30 and 40°, guaranteeing the deviation from blank At, although it is not very noticeable. As a result, the AtPdNP sample was prepared with a double concentration of 2 mM palladium chloride (Fig. 11c). Increasing amounts of palladium were clearly observed between 30 and 40°, and a noticeable hump-shaped peak was seen in the sample analysis.

The noticeable colour difference between the catalysts made from 1 mM and 2 mM palladium solutions was also observed (Fig. 8d).

#### 3.6.7 Scanning electron microscopy (SEM)

**Fig. 12:**
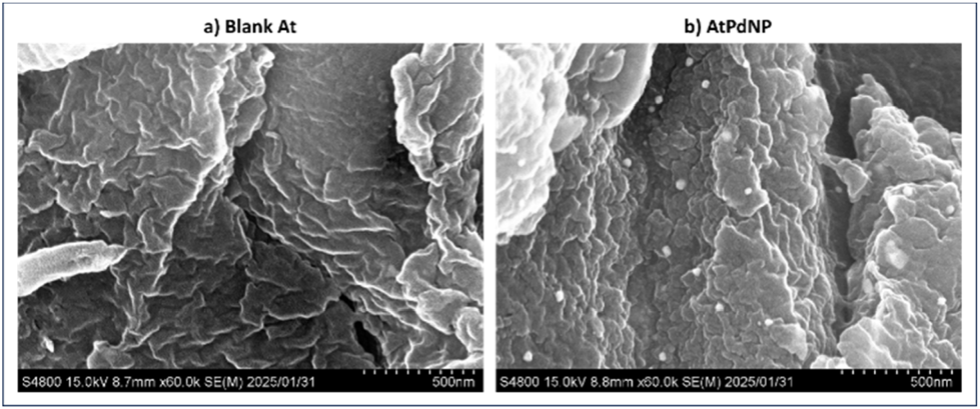
SEM images of (a) blank At and (b) AtPdNP catalyst.

A scanning electron microscopy (SEM) study was performed to analyse the surface morphology of the AtPdNP catalyst (Fig. 12). As palladium was not doped into the culture, blank At was used as a reference (Fig. 12a). This sample does not show any dots in the SEM image, confirming the absence of PdNPs. However, on the other hand, in the SEM image of AtPdNP catalyst, where palladium was doped in the culture, a number of small dots are easily visible, confirming the doping of palladium nanoparticles.

Also, due to the presence of palladium, the surface of AtPdNP was much smooth compared to blank biomass.

#### 3.6.8 Transmission electron microscopy (TEM)

**Fig. 13:**
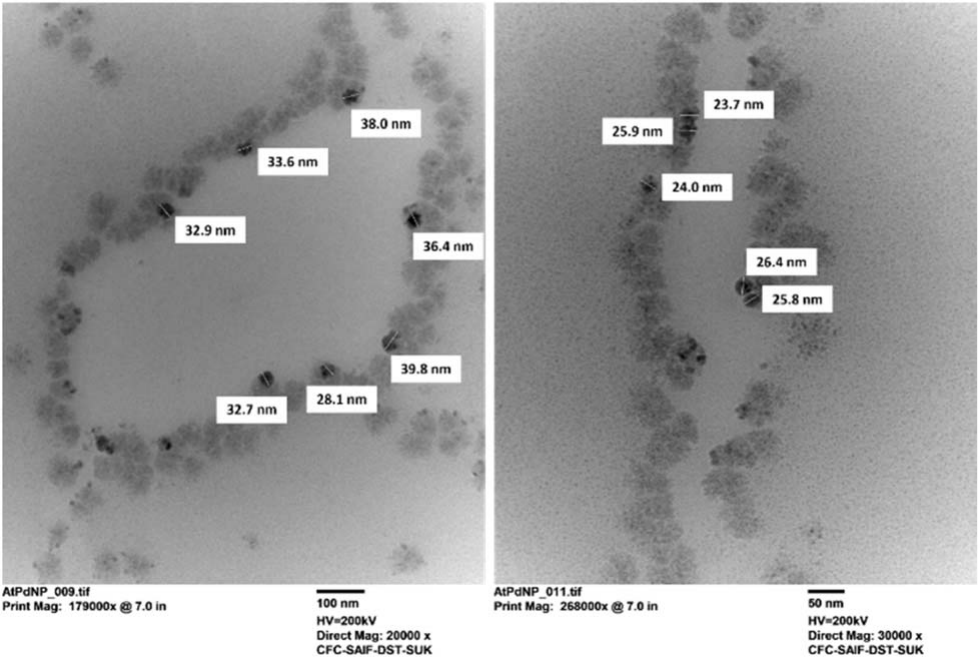
TEM images of AtPdNP.

TEM analysis was used to study the bulk morphology and size distribution of palladium nanoparticles (AtPdNP) produced by *Aspergillus trinidadensis*. The TEM images demonstrated that the palladium nanoparticles were successfully doped and stabilised by the biomass of *Aspergillus trinidadensis* (Fig. 13).

**Fig. 14:**
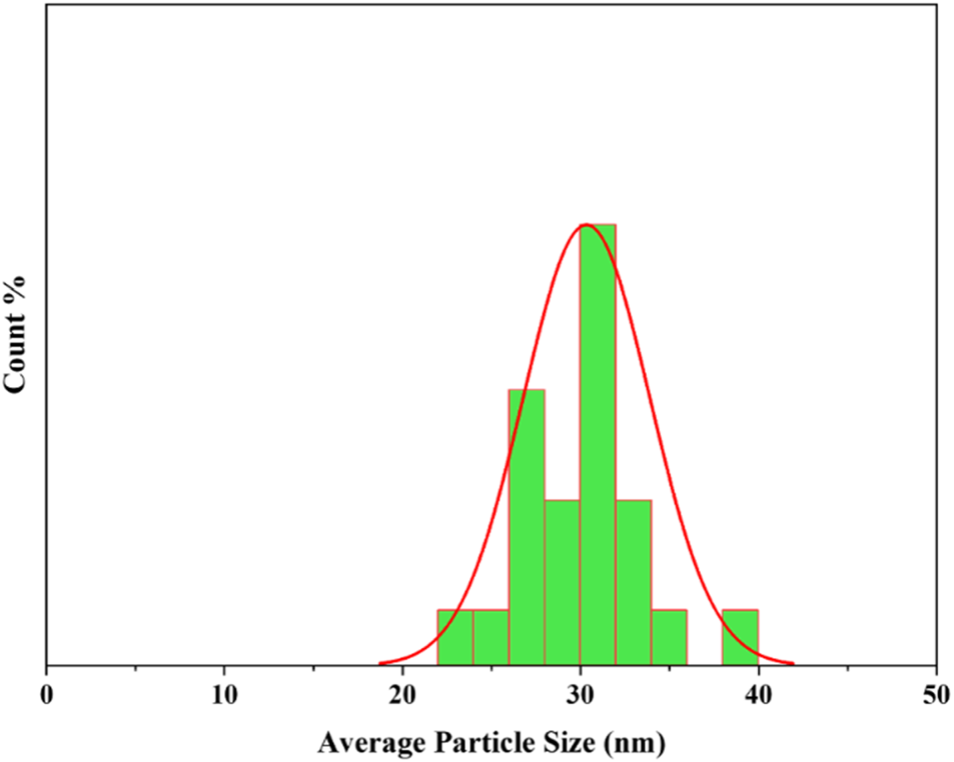
Size distribution curve of AtPdNP.

The TEM SAED pattern indicated that the sample was amorphous overall, which may be due to the extremely low concentration of palladium nanoparticles (atomic percentage of 0.01%), which was also validated by TEM-EDX. While metals exhibit a crystalline nature, their amorphous form was due to extremely low palladium concentration and relatively very high concentration of cell components. The examination on the nano scale has shown that the palladium nanoparticles became apparent in the size range from 20 to 40 nm. The TEM analysis clearly demonstrated the successful doping of palladium nanoparticles with biological moieties of *Aspergillus trinidadensis*. The particle size distribution graph (Fig. 14) displayed a Gaussian shape, and the average particle diameter was 30.33 ± 3.56 nm.

The observed size range was between 20 nm and 40 nm, indicating a relatively limited size distribution suitable for catalytic applications.

#### 3.6.9 Energy dispersive X-ray (EDX) spectroscopy and colour mapping

**Fig. 15:**
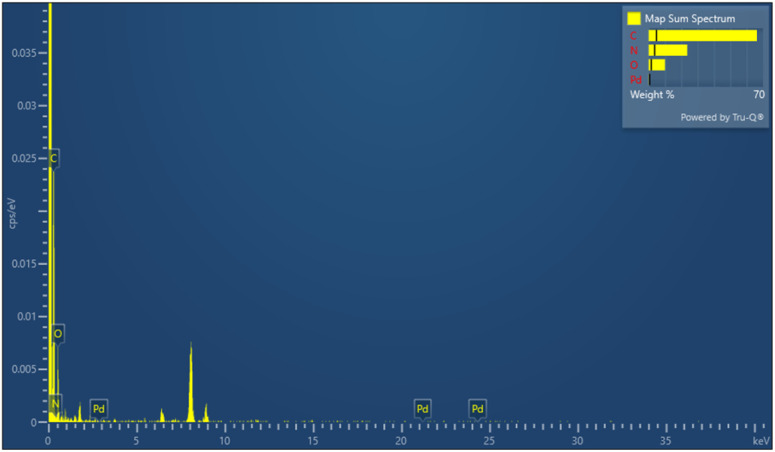
EDX spectrum of AtPdNP.

Energy Dispersive X-ray (EDX) spectroscopy was employed to analyse the elemental composition of the synthesized palladium nanoparticles as a heterogeneous catalyst. As shown in the EDX spectrum without grid (Fig. 15), characteristic signals for palladium (Pd) were detected, along with peaks for carbon (C), nitrogen (N), and oxygen (O). These additional elements certainly originated from the biological components or natural capping agents introduced during the green synthesis process.

According to the elemental analysis data (Table 6), palladium was present at an atomic percentage of 0.01%, confirming its successful incorporation into the nanostructure.

**Table 6:** EDX map sum spectrum of AtPdNP

Sr. no.	Element	Line type	*k* factor	Absorption correction	Wt%	Wt% sigma	Atomic%
1	C	K series	2.787	1.00	66.31	4.76	70.50
2	N	K series	3.538	1.00	23.58	3.61	21.50
3	O	K series	2.033	1.00	10.01	1.61	7.99
4	Pd	K series	8.754	1.00	0.10	0.62	0.01
	Total				100.00		100.00

**Fig. 16:**
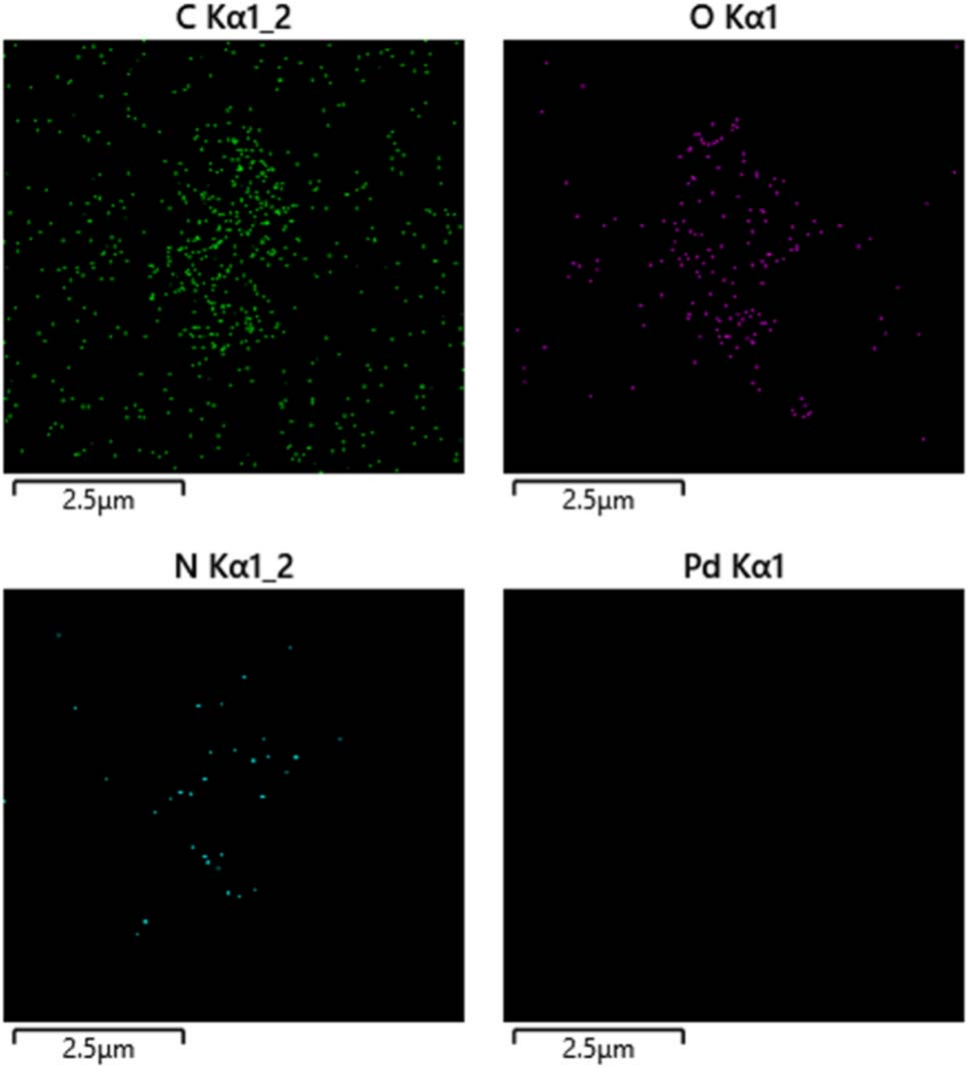
EDX colour mapping of AtPdNP.

The dominant presence of carbon (70.50%), nitrogen (21.50%) and oxygen (7.99%) points to the involvement of biomolecules derived from the fungal extract used as a reducing and stabilizing agent. The colour mapping of AtPdNP is displayed in Fig. 16, where colour dots represent carbon, nitrogen and oxygen. Palladium element was not observed much because of its extremely low concentration. Overall, the EDX results supported the effective biosynthesis of the Pd nanoparticles and offered insight into the surface chemistry influenced by the green synthesis method.

#### 3.6.10 HPLC (high-performance liquid chromatography)

The conversion of nitrobenzene to aniline was monitored by HPLC. The HPLC chromatogram of the synthesized aniline displayed a single, sharp peak at a retention time (*R*_*t*_) of 7.886 minutes, which matched with the retention time of the authentic reference standard aniline. This confirmed the successful formation of the target product. In contrast, the starting material nitrobenzene exhibited a major peak at (*R*_*t*_) 10.827 minutes, indicating that the product is chemically distinct from the precursor. The purity of the synthesized aniline, based on the peak area, was found to be 96.74%, signifying minimal presence of impurities. This chromatographic analysis was carried out on a C18 reverse-phase column (4.6 × 250 mm, 5 µm) using a mobile phase of acetonitrile and water (70 : 30, v/v) under isocratic conditions at a flow rate of 0.5 mL min^−1^ and a detection wavelength of 254 nm. The consistency in retention time with the standard and the absence of the starting compound peak confirmed the complete conversion and high purity of the final product (Fig. S3 and Table S3).

## 4. Conclusion

Here, we have demonstrated *Aspergillus trinidadensis* as an efficient microbe for the biosynthesis of AtPdNP. A one-pot method that is sustainable, eco-friendly and efficient has been established. UV-vis, FT-IR, TGA-DSC-DTA, XPS, SEM, TEM, EDX, elemental mapping, powder XRD and zeta potential analysis were all used to fully characterize the morphologies of AtPdNP. All these analyses confirmed the formation of palladium nanoparticles with uniform size distribution from 20 to 40 nm. Also, it confirmed the efficient capping of palladium nanoparticles by biomass. Nitrobenzene reduction was used to demonstrate the use of the produced catalyst. As a catalytic investigation, AtPdNP produced under various circumstances was employed. The highest reduction efficiency was demonstrated by AtPdNP synthesized with a 160 g mL^−1^ biomass concentration and a 36 hour culture age under 24 hour incubation at 34 °C.

## Author contributions

All the experiments were designed and performed by Hemal B. Rathod and Paresh N. Patel. The spectral study, interpretation, and correlations were done by Hemal B. Rathod, Amar G. Deshmukh, Yashasvi N. Desai and Paresh N. Patel. The manuscript was written by Hemal B. Rathod. The final proofreading and editing were done by Paresh N. Patel.

## Conflicts of interest

The authors declare that they have no known competing financial interests or personal relationships that could have appeared to influence the work reported in this paper.

## Supplementary Material

NA-OLF-D6NA00174B-s001

## Data Availability

Additional data related to main manuscript are available in the supplementary information (SI). Supplementary information is available. See DOI: https://doi.org/10.1039/d6na00174b.
