# Supplementary material for: A palladium bio-nanocomposite as an efficient heterogeneous catalyst for nitro reduction: a fungus mediated green and sustainable process
Source: Nanoscale Adv. 2026 May 28;8(14):3981–92. doi: 10.1039/d6na00174b (PMC13274705; doi:10.1039/d6na00174b)
Supplement: NA-008-D6NA00174B-s001 [file NA-008-D6NA00174B-s001.pdf]

Supplementary Information (SI) for Nanoscale Advances.
This journal is © The Royal Society of Chemistry 2026

Palladium Bio-Nanocomposite as an Efficient Heterogeneous Catalyst for Nitro Reduction: Fungus Mediated Green and Sustainable Process

Hemal B. Rathod, Amar G. Deshmukh, Yashasvi N. Desai, Paresh N. Patel*

Laboratory of Bio-Organic Chemistry, Tarsadia Institute of Chemical Science (TICS), Uka Tarsadia University, Bardoli – 394350, Gujarat, India.

1. Calculation of TON and TOF

- For each reaction, 0.005 g of AtPdNP (catalyst) is utilized for the 0.001 Mole nitro reduction. At the time of nanoparticle synthesis, 1 gram of AtPdNP (catalyst) Batch is prepared.
- The molecular weight of $PdCl_2$, which is utilized as a metal precursor, is 177.33 g/mol, while the molecular weight of Pd alone is 106.42 g/mol.

$$1\ M \rightarrow 1000\ mL\ distilled\ water \rightarrow 106.42\ g\ Pd$$
$$0.001\ M \rightarrow 1000\ mL\ distilled\ water \rightarrow\ ?(x)\ g\ Pd$$
$$x = \frac{0.001\ M \times 106.42\ g}{1\ M}$$
$$x = 0.106\ g\ Pd\ in\ 1000\ mL\ 0.001\ M\ solution$$

- 1 g of AtPdNP (catalyst) is prepared in a 500 mL 0.001 M $PdCl_2$ solution

$$0.001\ M \rightarrow 1000\ mL\ distilled\ water \rightarrow 0.106\ g\ Pd$$
$$0.001\ M \rightarrow 500\ mL\ distilled\ water \rightarrow\ ?(y)\ g\ Pd$$
$$y = \frac{500\ mL \times 0.106\ g}{1000\ mL}$$
$$y = 0.053\ g\ Pd\ in\ 1\ g\ AtPdNP$$

- A 1 g AtPdNP (catalyst) batch is prepared using 0.053 g Pd; only 0.005 g of AtPdNP (catalyst) is used in the reaction.

$$1\ g\ AtPdNP \rightarrow 0.053\ g\ Pd$$
$$0.005\ g\ AtPdNP \rightarrow\ ?\ (z)\ g\ Pd$$
$$z = \frac{0.005\ g \times 0.053\ g}{1\ g}$$
$$z = 0.000265\ g\ Pd\ in\ 0.005\ g\ AtPdNP$$

- Mole calculation of Pd in the reaction

$$106.42\ g\ Pd \rightarrow 1\ Mole$$
$$0.000265\ g\ Pd \rightarrow\ ?(X)\ Mole$$
$$X = \frac{0.000265\ g \times 1\ Mole}{106.42\ g}$$
$$X = 0.0000024\ Mole\ Pd\ used\ in\ reaction$$

➢ Calculation of TON and TOF

$$TON = \frac{Number\ of\ Moles\ of\ Reactant\ Conversion}{Moles\ of\ Catalyst\ Active\ Centres}$$

$$TON = \frac{0.001\ Mole}{0.0000024\ Mole}$$

$$TON = 416.66$$

$$TOF = \frac{TON}{Reaction\ Time\ in\ Hours}$$

$$TOF = \frac{416.66}{0.5\ hr}$$

$$TOF = 833.32\ hr^{-1}$$

2. Zeta Size and Zeta Potential

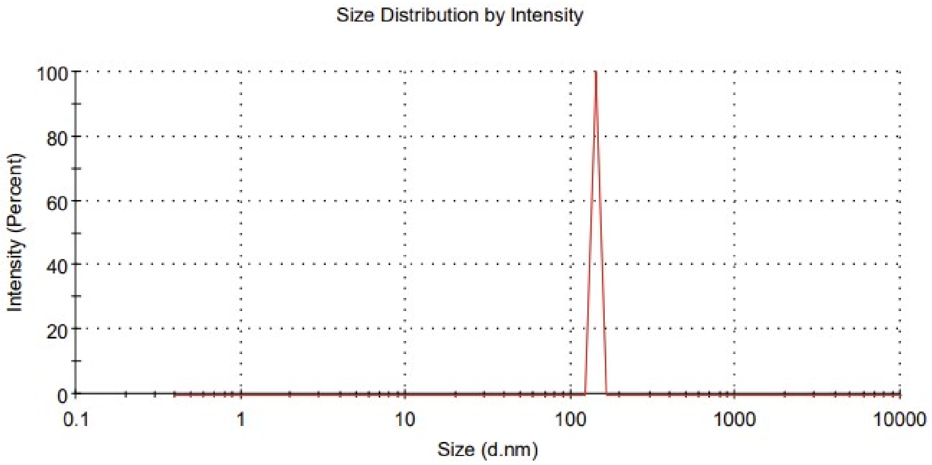

Figure S1: Zeta size for AtPdNP

Table S1:

Zeta size for AtPdNP

			Size (d.nm)	% Intensity	St Dev (d.nm)
**Z-Average (d.nm):**	1452	**Peak 1:**	141.8	100.0	1.907e-6
**PdI:**	1.000	**Peak 2:**	0.000	0.0	0.000
**Intercept:**	1.40	**Peak 3:**	0.000	0.0	0.000

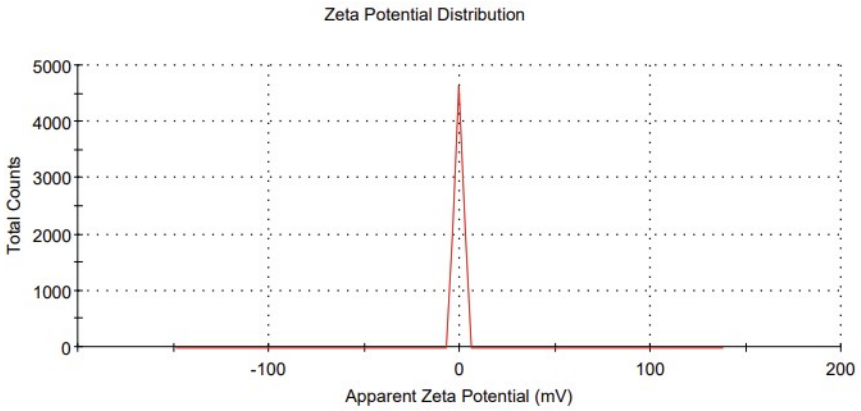

Figure S2: Zeta potential for AtPdNP

Table S2:

Zeta potential for AtPdNP

			Mean (mV)	Area (%)	St Dev (mV)
**Zeta Potential (mV):**	1452	**Peak 1:**	141.8	100.0	1.907e-6
**Zeta Deviation (mV):**	1.000	**Peak 2:**	0.000	0.0	0.000
**Conductivity (mS/cm):**	1.40	**Peak 3:**	0.000	0.0	0.000

3. High-Performance Liquid Chromatography (HPLC)

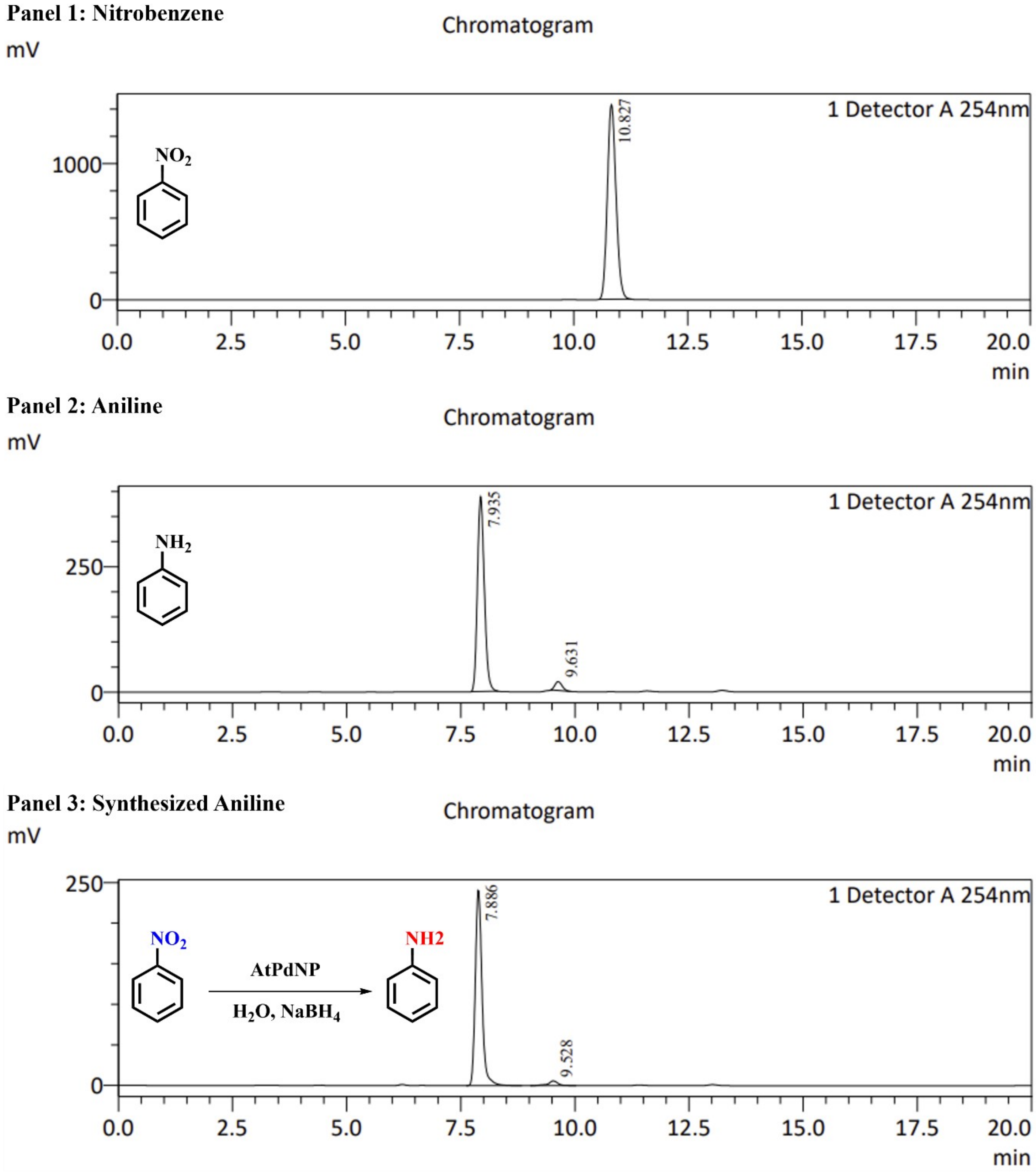

Figure S3: HPLC chromatogram of (Panel 1) standard reference nitrobenzene, (Panel 2) standard reference aniline, (Panel 3) synthesized aniline

Table S3:

Name	Peak#	Ret. Time	Area	Area %
**(Panel 1) HPLC Peak Table for Standard Reference Nitrobenzene**				
Nitrobenzene	1	10.827	18871343	100.00
	**Total**		**18871343**	**100.00**
**(Panel 2) HPLC Peak Table for Standard Reference Aniline**				
Aniline	1	7.935	4090000	95.449
Impurity	2	9.631	194997	4.551
	**Total**		**4284997**	**100.00**
**(Panel 3) HPLC Peak Table for Synthesized Aniline**				
Aniline	1	7.886	2448607	96.746
Impurity	2	9.528	82351	3.254
	**Total**		**2530958**	**100.00**

4. ICP-OES

➢ A 1 g AtPdNP (catalyst) batch is prepared using 0.053 g Pd; only 0.050 g of AtPdNP (catalyst) is used in the sample preparation for ICP-OES in 5 mL of distilled water.

$$1\ g\ AtPdNP \rightarrow 0.053\ g\ Pd$$
$$0.050\ g\ AtPdNP \rightarrow ?\ (z)\ g\ Pd$$
$$z = \frac{0.050\ g\ \times 0.053\ g}{1\ g}$$
$$z = 0.00265\ g\ Pd\ in\ 0.050\ g\ AtPdNP$$

5. Comparison of AtPdNP catalyst performance with other known catalysts

Table S1: Comparison of AtPdNP catalyst performance with other known catalysts.

Entry	Catalyst	Solvent	Reducing Agent	Temp. ^{O}C	Time (min)	Yield (%)	Ref.
1	Pd@PANI	H_2O	$NaBH_4$	RT	240	92	1
2	Pd@NP	H_2O: EtOH	$NaBH_4$	RT	120	-	2
3	MNPs@PIL@AuNPs	H_2O	$NaBH_4$	RT	70	96	3
4	AuPd NPs	H_2O	$NaBH_4$	60	180	99.4	4
5	Bio-Pd	H_2O	$NaBH_4$	RT	60	95	5
6	Pt/$NaBH_4$	EtOH	$NaBH_4$	RT	60	88.8	6
7	CuNP/WS-1	H_2O	$NaBH_4$	35	240	88	7
8	Fe3O4@sepiolite-Pd^{2+}	EtOH: H_2O	$NaNH_4$	70	50	85	8
9	AtPdNP	H_2O	$NaBH_4$	RT	30	98.80	This Work

Reference

1. Wang, G., Yuan, S., Wu, Z., Liu, W., Zhan, H., Liang, Y., ... & Bi, S. (2019). Ultra-low-loading palladium nanoparticles stabilized on nanocrystalline Polyaniline (Pd@ PANI): a efficient, green, and recyclable catalyst for the reduction of nitroarenes. Applied Organometallic Chemistry, 33(11), e5159.
2. Mekkaoui, A. A., Jennane, S., Aberkouks, A., Boualy, B., Mehdi, A., Ait Ali, M., ... & El Houssame, S. (2019). Palladium nanoparticles supported on mesoporous natural phosphate: an efficient recyclable catalyst for nitroarene reduction. *Applied Organometallic Chemistry*, *33*(10), e5117.
3. Moghaddam, F. M., Ayati, S. E., Firouzi, H. R., Hosseini, S. H., & Pourjavadi, A. (2017). Gold nanoparticles anchored onto the magnetic poly (ionic-liquid) polymer as robust and recoverable catalyst for reduction of Nitroarenes. *Applied Organometallic Chemistry*, *31*(12), e3825.
4. de Souza, A. P. N., da Silva, G. F. S., CS Santos, E., de Gois, J. S., Mendoza, C. A. D., de Barros, S. D. T., ... & Senra, J. D. (2026). Eco-Friendly Synthesis of AuPd Bimetallic Nanoparticles Using Alpinia zerumbet for Efficient Reduction of Nitro Compounds. *ACS Omega*.
5. Xie, J., Egan-Morriss, C., Coker, V. S., Sullivan-Allsop, S., Cai, R., Haigh, S. J., & Lloyd, J. R. (2026). Microbial synthesis of bimetallic Pd–Rh and Pd–Pt nanoparticle catalysts. *Nanoscale Advances*.
6. Long, Y., Yuan, B., Niu, J., Tong, X., & Ma, J. (2015). Distinctive size effects of Pt nanoparticles immobilized on Fe_3O_4 @ PPy used as an efficient recyclable catalyst for benzylic alcohol aerobic oxidation and hydrogenation reduction of nitroaromatics. *New Journal of Chemistry*, *39*(2), 1179-1185.
7. A. Zamani, A. Poursattar Marjani, A. Nikoo, M. Heidarpour, A. Dehghan, Inorg. Nano-Metal Chem. 2018, 48, 176.
8. Ghonchepour, E., Islami, M. R., Bananezhad, B., Mostafavi, H., & Tikdari, A. M. (2019). Synthesis of recoverable palladium composite as an efficient catalyst for the reduction of nitroarene compounds and Suzuki cross-coupling reactions using sepiolite clay and magnetic nanoparticles (Fe_3O_4@ sepiolite-Pd^{2+}). *Comptes Rendus. Chimie*, *22*(1), 84-95.
